# Carrier-Free Cisplatin–Dactolisib Nanoparticles for Enhanced Synergistic Antitumor Efficacy

**DOI:** 10.1021/acsbiomaterials.4c00672

**Published:** 2025-02-24

**Authors:** Mei Zhang, Qiuxia Tan, Sevil Gonca, Minhuan Lan, Bin-Zhi Qian, Xianfeng Chen, Norbert Radacsi

**Affiliations:** † School of Engineering, Institute for Materials and Processes, University of Edinburgh, Robert Stevenson Road, Edinburgh EH9 3FB, U.K.; ‡ School of Engineering, Institute for Bioengineering, University of Edinburgh, The King’s Buildings, Edinburgh EH9 3JL, U.K.; § Key Laboratory of Hunan Province for Water Environment and Agriculture Product Safety, College of Chemistry and Chemical Engineering, Central South University, Changsha 410083, China; ∥ Medical Research Council Centre for Reproductive Health, College of Medicine and Veterinary Medicine, Queen’s Medical Research Institute University of Edinburgh, Little France Crescent, Edinburgh EH16 4TJ, U.K.; ⊥ Fudan University Shanghai Cancer Center, Department of Oncology, Shanghai Medical College, The Human Phenome Institute, Zhangjiang-Fudan International Innovation Center, Fudan University, Shanghai 200433, China

**Keywords:** drug delivery, antitumor agents, pure drug nanoparticles, combination therapy, self-assembly

## Abstract

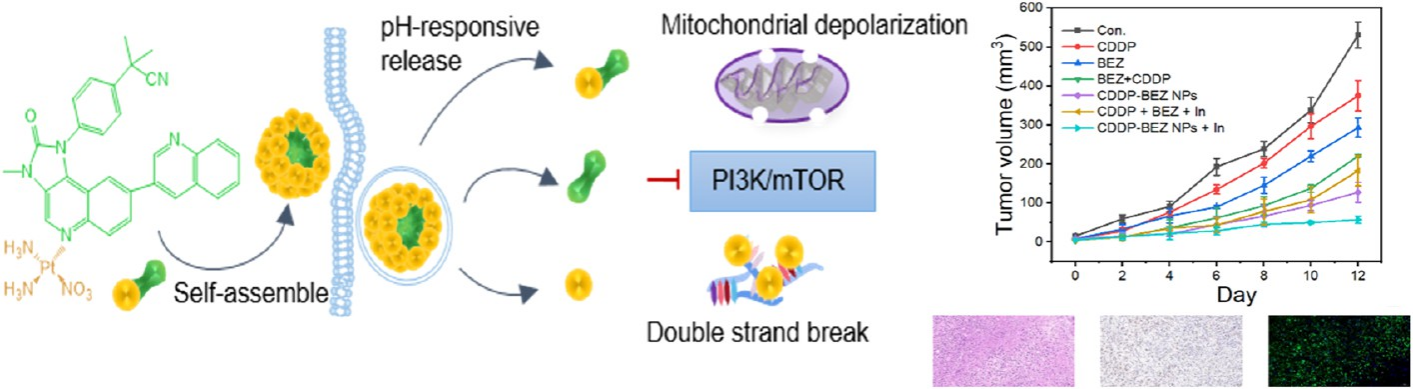

Cisplatin (CDDP) is one of the most commonly used chemotherapeutic agents for solid tumors and hematologic malignancy. However, its therapeutic outcomes have remained unsatisfactory due to severe side effects, a short elimination half-life, the emergence of drug resistance, and the induction of metastasis. Combination with other chemotherapeutic agents has been proposed as one strategy to address the drawbacks of CDDP-based therapy. Therefore, this study aimed to boost the antitumor efficacy of cisplatin (CDDP) with a PI3K/mTOR dual inhibitor, dactolisib (BEZ), via a carrier-free codelivery system based on the self-assembly of the coordinated CDDP–BEZ. The synthesized CDDP–BEZ nanoparticles (NPs) possess sensitive pH-responsiveness, facilitating the delivery of both drugs to cancer cells. CDDP–BEZ NPs specifically enhanced cytotoxicity in cancer cells due to the synergy between cisplatin and dactolisib, resulting in augmented DNA damage, activation of mitochondria-dependent apoptosis, and increased inhibition on the PI3K/mTOR signaling axis. The inhibition of tumor migration and metastasis by CDDP–BEZ NPs was observed both in vitro and in vivo. Our data suggest that CDDP–BEZ NPs could serve as a safe and effective platform to maximize the synergy between both drugs in combating cancer, presenting a strategy to promote the therapeutic efficacy of platinum-based chemotherapeutic agents by combining them with PI3K inhibitors.

## 1 Introduction

Cancer, a collection of diseases with rapid proliferation of abnormal cells, with the possibility of invading the whole body, contributes to the second-highest number of global deaths. According to a report of the World Health Organization (WHO), cancer led to approximately 9.6 million deaths in 2018.^1^ Cisplatin (CDDP) has remained one of the most commonly used chemotherapeutic agents for solid tumors and hematologic malignancy. By covalently binding to purine bases, guanine and adenine, CDDP could induce double-strand breaks (DSBs) of DNA, resulting in the apoptotic or nonapoptotic death of rapidly proliferative cells.^2^ Despite the widespread clinical application of CDDP, the therapeutic outcomes remained unsatisfactory due to the occurrence of severe side effects, including nephrotoxicity and cardiotoxicity.^2^ Besides, the relatively short elimination half-life, the emergence of drug resistance, and the induction of metastasis also contributed to the limited therapeutic outcomes of CDDP, calling for the development of a novel therapeutic strategy.^3–5^

New therapeutic strategies aim to identify methods with superior efficacy, reduced dose-dependent toxicity, lack of cross-resistance, or improved pharmacological properties compared to the traditional approach of CDDP monotherapy. Monotherapeutic techniques nonselectively target actively proliferating cells, ultimately destroying both healthy and cancerous cells. CDDP is often used in combination with multiple drugs so that the drugs work together in a synergistic or additive way, requiring a lower therapeutic dose of each individual drug. Moreover, the process of developing a new cancer drug is costly and extremely time-consuming, and 5 year survival rates for most metastatic cancers are still quite low. Therefore, a combination with other chemotherapeutic agents has been proposed as a strategy to provide efficient and effective results at an affordable cost and to overcome the disadvantages of CDDP-based therapy.^6,7^ Emerging evidence suggested the dominant role of the hyperactive PI3K/AKT/mTOR axis in the occurrence of resistance to CDDP, with the downregulation of pro-apoptotic proteins, the promotion of epithelial–mesenchymal transition, and the upregulation of multidrug resistance proteins.^7−10^ Therefore, the inhibitors for this signaling pathway have been exploited to resensitize cancer cells to therapy with CDDP. However, the difference between CDDP and most PI3K/AKT inhibitors in solubility, biodistribution, and the membrane transportation pathway hinders the realization of the maximum synergism, increasing the difficulties in the design of dosage and schedule for a proper regimen.^7^ The third-generation PI3K/mTOR inhibitor dactolisib (BEZ) was developed to provide more complete inhibition of multiple signaling points in the pathway, and many studies have shown that BEZ, when used in combination with anticancer drugs such as cisplatin, doxorubicin, and paclitaxel, inhibits tumor growth and induces synergistic antitumor effects in cancer cells compared to monotherapy with these drugs alone.

The rapid development of nanotechnology not only extends the possibilities of deploying various materials for photodynamic therapy in anticancer practice but also makes nanodrug delivery systems (nanoDDSs) promising candidates for the efficient codelivery of therapeutic agents to the target site.^11−13^ NanoDDSs are divided into two groups: carrier-assisted drug delivery systems (CDDSs) and carrier-free nanodrugs. CDDSs have been widely used in the treatment of cancer due to their ability to improve the biological stability and bioavailability of therapeutic agents. Despite the clinical success of some CDDSs (e.g., Doxil and Genexol-PM) in cancer treatment, challenges such as the undesirable drug loading capacity of the carriers and potential systemic toxicity from the substances used as carriers limit their clinical translation. Moreover, the process of carrier design and synthesis is relatively complex, leading to batch-to-batch variation in the drug metabolic process and therapeutic effects of carrier-based nanomedicines. With these problems in mind, a great deal of research has been devoted to the development of carrier-free, pure drug self-delivery systems on the basis of drug–drug conjugates. With hydrophobic interaction, electrostatic interaction, hydrogen bonding, or aromatic ligand stacking as the driving force for the particle formation, the carrier-free self-assemblies presented an encouraging approach for drug delivery because they do not require excipient materials and thus can overcome the potential problems raised by vector-based nanoparticles (NPs), including insufficient drug loading efficiency and safety concerns.^14,15^ Overall, carrier-free pure nanodrug delivery systems are a promising therapeutic strategy to increase efficacy and reduce side effects compared to free drugs. However, inadequate physical stability in blood circulation and uncontrollable drug release in tumor sites remained challenges for most of the reported pure drug NPs, necessitating the design and fabrication of stimuli-responsive pure drug NPs.^16^

**Scheme 1:**
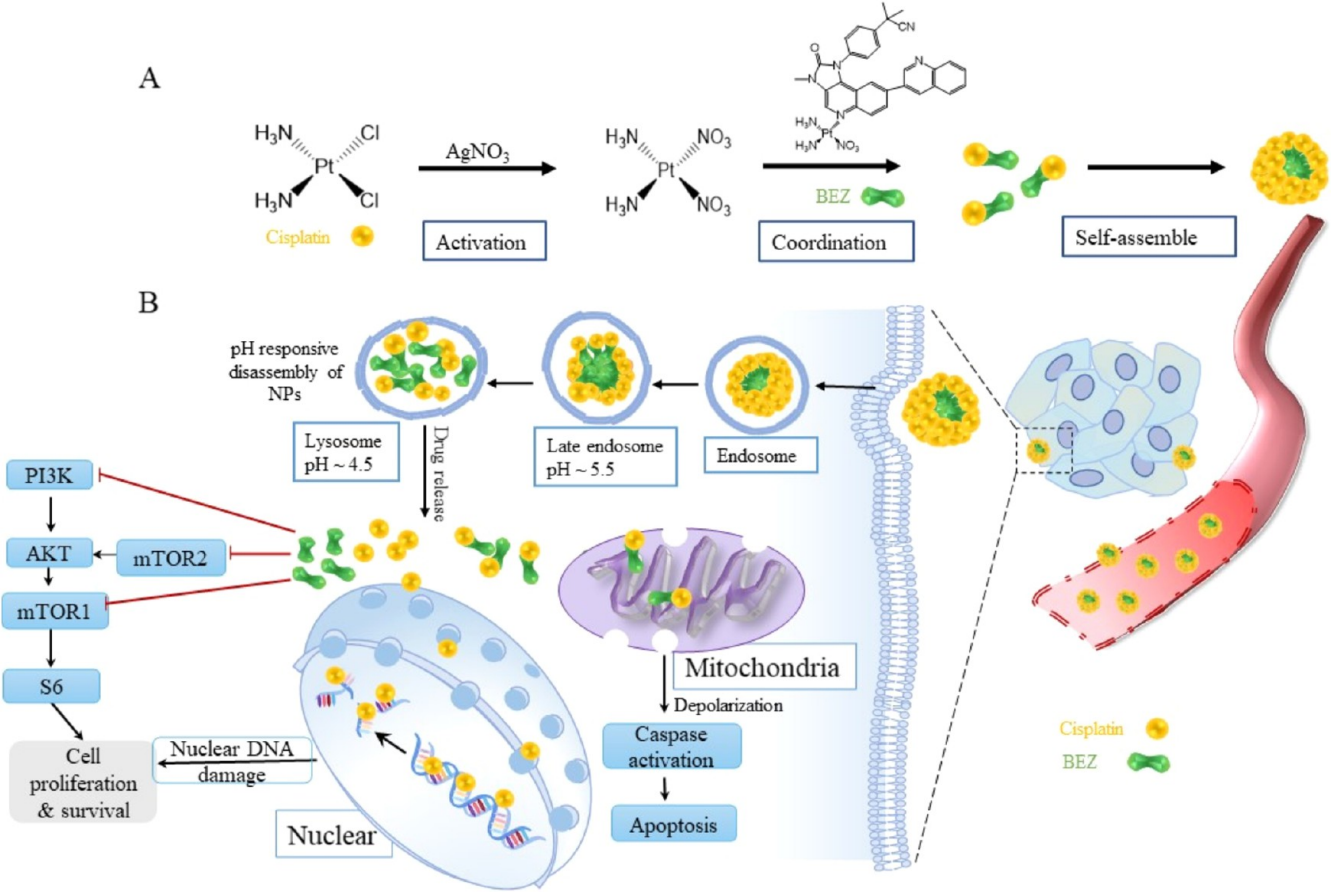
Schematic Illustration of the Fabrication and the Antitumor Mechanism of CDDP–BEZ NPs (A) CDDP and BEZ were conjugated through a coordination bond, after which the conjugate self-assembled into NPs driven by its own amphiphilicity. (B) After the entry to the cancer cell, CDDP–BEZ NPs could release free CDDP and BEZ upon the acidic environment in the lysosome, after which both CDDP and BEZ would exert the antitumor efficacy on the respective functional sites. The coordinated CDDP–BEZ would induce mitochondria-dependent apoptosis.

Here, we established a self-assembled carrier-free nanoDDS for the codelivery of CDDP and dactolisib (BEZ, a PI3K/mTOR dual inhibitor), expecting to achieve a boosted synergistic antitumor efficacy. A drug–drug conjugate, CDDP–BEZ, was first synthesized by a coordination bond between the platinum (Pt) of CDDP and the nitrogen (N) atom on the quinoline moiety of BEZ, followed by self-assembly into uniform NPs because of the amphiphilic nature of the conjugate (Scheme 1). The as-prepared nanoDDS implemented a way to precisely deliver both CDDP and BEZ into the same cell with high efficiency, followed by a synergistic antitumor efficacy specifically enhanced in cancer cells due to the pH-responsiveness of the coordination bond to the lower lysosomal pH value. By simultaneously affecting DNA and inhibiting the PI3K signaling axis, CDDP–BEZ NPs exhibited a potent antiproliferative effect on cancer cells. Besides, the increased lipophilicity due to the coordination with hydrophobic BEZ facilitated the accumulation of CDDP in mitochondria, resulting in the activation of mitochondria-dependent intrinsic apoptosis and subsequently leading to a further elevated synergistic efficacy in killing cancer cells. Additionally, CDDP–BEZ NPs successfully restrained the cell migration and invasion induced by chemotherapy, which was observed in both two-dimensional (2D) cell culture and three-dimensional (3D) tumor spheroids. The results of the in vivo study also proved that CDDP–BEZ NPs could function prominently even under the complicated physiological environment, constraining the progression of tumors in mice without causing obvious safety issues. To the best of our knowledge, this study was the first to demonstrate the feasibility to codeliver a Pt-based chemotherapeutic agent and a PI3K/mTOR dual inhibitor into cancer cells without introducing any foreign materials. Our approach represents a safe and effective platform to tackle the existing hindrance in the combination therapy of CDDP and PI3K/mTOR dual inhibitors in the hope of achieving further industrial and clinical application for enhanced therapeutic outcomes in treating solid tumors.

## 2 Materials and Methods

### 2.1 Materials and Reagents

All chemicals were used as purchased without any further purification. Cisplatin (CDDP) was purchased from Cayman Chemical (MI). Dactolisib (BEZ) was purchased from Stratech Scientific (Cambridge, U.K.). Silver nitrate (AgNO_3_), *N*,*N*-dimethylformide (DMF), and crystal violet were purchased from Sigma-Aldrich (St Louis). Anti-γ-H2A.X antibody, anticleaved caspase-3 antibody, anticleaved caspase-9 antibody, anti-S6 ribosomal protein antibody, antiphospho-S6 ribosomal protein antibody, anti-AKT (pan) antibody, antiphospho-AKT antibody, goat anti-rabbit IgG, and goat anti-rabbit IgG (H + L) (Alexa Fluoro488) were purchased from Cell Signaling Technology (MA). The JC-1 mitochondrial membrane potential (MMP) assay kit was purchased from Abcam (Cambridge, U.K.). The Calcein-AM/PI live–dead cell staining kit was purchased from APExBIO Technology (Boston). Matrigel Matrix was purchased from Corning Inc. (New York).

### 2.2 Synthesis of the Coordinated CDDP and BEZ and the Preparation of Pure Drug NPs

The coordination between CDDP and BEZ was performed using the following procedures. Briefly, CDDP (15 mg, 0.05 mmol) was first activated by AgNO_3_ (17 mg, 0.1 mmol) in 10 mL of DMF at room temperature in darkness for 12 h, whereafter the resultant silver chloride was removed by centrifugation as the precipitation. The coordination reaction was then performed by mixing the supernatant and 23.5 mg of BEZ (0.05 mmol) dissolved in 10 mL of DMF at 60 °C in darkness for 24 h. The NPs were subsequently formed by diluting the reaction solution with DI water of 3-fold the volume, followed by dialysis against deionized water (molecular weight cutoff (MWCO) = 3.5 kDa) for 4 h to remove any unreacted organic molecules. The as-prepared nanoparticles were stored in a fridge at 4 °C in darkness. The original molar ratio of cisplatin and BEZ on a single NP was calculated to be 2:3.

### 2.3 Physicochemical Characterization of Coordinated CDDP and BEZ

The particle size and ζ potential of the as-prepared NPs were measured at 25 °C by dynamic light scattering (DLS, ZetaPAL, Brookhaven). Transmission electron microscopy (JEOL TEM-1400 Plus) was used to observe the morphology of the as-prepared NPs. The content of CDDP and BEZ in the coordinated conjugate or NPs was measured by inductively coupled plasma (ICP) and a UV–vis spectrometer, respectively, by which the molar ratios between CDDP and BEZ could be calculated.

### 2.4 Mechanism of Particle Formation of CDDP–BEZ NPs

CDDP–BEZ NPs were incubated with urea, Tween 20, Triton X-100, or NaCl at different concentrations for 30 min, respectively, and then the particle size was measured by DLS. The morphology of CDDP–BEZ NPs after incubation with 100 mM of the above-mentioned chemicals was observed by TEM.

### 2.5 Storage Stability and pH-Responsiveness of CDDP–BEZ NPs

To evaluate the storage stability of CDDP–BEZ NPs, the particle size was monitored by DLS once a week for 2 months. The morphological change induced by different pH values was observed by TEM after incubation in 1× phosphate-buffered saline (PBS) of different pH values (7.4, 6.5, and 5.0) for 1, 4, and 8 h.

The in vitro stimuli-responsive drug release profile was explored by the dialysis membrane method. Briefly, 3 mL of NP solution was transferred into dialysis tubes, which were then immersed into 27 mL 1× PBS of different pH values (7.4, 6.5, and 5.0). Tween 80 was added to the buffer to solubilize the released BEZ. The releasing media were incubated at 37 °C with a constant shaking of 120 rpm. For each releasing condition, samples of 1.5 mL were withdrawn from the releasing media at 1, 2, 3, 6, 9, 12, 24, 48, and 72 h and replenished with fresh releasing media of the same volume. The analysis at each time point was carried out in triplicates. The concentration of BEZ in the releasing media was calculated by UV–vis absorbance at 328 nm according to the established calibration. The concentration of cisplatin in the releasing media was calculated by the amount of Pt measured with ICP-mass spectrometry (ICP-MS).

### 2.6 Cell Culture and Cell Lines

Human breast cancer cell line MCF-7, MDA-MB-231, and human ovarian cancer cell line, NIH:OVCAR-3, human breast epithelial cell line MCF-10A, and human umbilical vein epithelial cell line HUVEC (ATCC, Manassas, VA) were used in the in vitro study. MCF-7 and MDA-MB-231 were cultured in Dulbecco’s modified Eagle’s medium containing 10% (v/v) fetal bovine serum (FBS). MCF-10A was cultured in Dulbecco’s modified Eagle’s medium containing 10% FBS and 0.01 mg/mL insulin. NIH:OVCAR-3 cells were cultured in RPMI 1640 media containing 20% (v/v) FBS and 0.01 mg/mL insulin. HUVECs were cultured in Endothelial Cell Growth Media-2 (EGM-2) containing 10% (v/v) FBS and VEGF. All cells were incubated in an incubator with a humidified atmosphere containing 5% CO_2_ at 37 °C. Cells were split using trypsin/ethylenediaminetetraacetic acid (EDTA) medium for passage or further in vitro study when 80% confluency was reached.

### 2.7 In Vitro Cytotoxicity Study

The in vitro cytotoxicity of CDDP–BEZ NPs was evaluated by the 3-[4,5-dimethylthiazol-2-yl]-2,5-diphenyltetrazolium bromide (MTT) assay according to the manufacturer’s instruction. MCF-7, MDA-MB-231, and OVCAR-3 cells were used for the anticancer activity study, and MCF-10A and HUVECs were used to investigate the cytotoxicity of different formulations on normal tissue cells. Cells were seeded into 96-well plates with a density of 1 × 10^4^ cells per well in 200 μL of culture media, followed by incubation for 24 h. The media were then replaced with FBS-free media containing CDDP–BEZ NPs at different concentrations. Cells treated by FBS-free media were used as blank control, and cells treated by BEZ, CDDP, or the mixture of dual drugs were studied as parallel control groups. After incubation for 24 or 72 h, 20 μL of MTT reagent was added to each well, followed by further incubation for another 4 h. The media were then removed, and 150 μL of dimethyl sulfoxide (DMSO) was added to each well to dissolve the formazan crystal formed in living cells. The optical densities (ODs) at 570 nm were measured with a microplate reader (BMG LABTECH, Germany). The combination index (CI) was calculated with CompuSyn according to the Chou–Talalay method. Each assay was repeated in triplicate.

The influence of different formulations on cell viability was then compared by a Calcein-AM/propidium iodide (PI) assay. Briefly, MCF-7 and MDA-MB-231 cells were seeded into a 6-well plate at a density of 3 × 10^5^ cells per well and incubated for 24 h before the assay. The media were then replaced with FBS-free media containing CDDP–BEZ NPs, CDDP, BEZ, or the mixture of dual drugs with a concentration of CDDP at 1 μg/mL. Cells treated with FBS-free media were evaluated as a blank control group. After incubation for 12 h, the cells were washed with PBS three times and then stained with a Calcein-AM/PI kit according to the manufacturer’s instructions. After the removal of excessive dye, the cells were observed, and the images were captured with an inverted fluorescence microscope (Nikon, Tokyo, Japan).

### 2.8 Cell Apoptosis Assay with Flow Cytometry

The Annexin V-FITC/PI assay was performed to assess the influence of different formulations on cellular apoptosis. Cells were seeded into a 12-well plate at a density of 1.5 × 10^5^ cells per well and incubated for 24 h before the assay. Cells were then treated with CDDP–BEZ NPs, CDDP, BEZ, or the mixture of dual drugs with a CDDP concentration of 1.5 μg/mL. After incubation for 12 h, the cells were trypsinized and collected in 400 μL of PBS, followed by rinsing with PBS twice. Thereafter, the cells were stained with the Annexin V-FITC/PI apoptosis detection kit according to the manufacturer’s protocols and then analyzed by a flow cytometer (Attune Nxt).

### 2.9 Wound Healing Assay

The influence of different formulations on the migration properties of MCF-7 and MDA-MB-231 was explored with a wound healing assay. Cells were seeded in 12-well plates at a density of 1.5 × 10^5^ cells per well. After incubation for 24 h, a 20 μL pipet tip was used to scratch the middle of the well along the diameter. The cells were then washed with PBS three times to remove floating cells and treated with CDDP–BEZ NPs, CDDP, BEZ, or the mixture of dual drugs with a CDDP concentration of 1 μg/mL. The cell healing was observed with a Nikon TE2000 inverted microscope after further incubation for 12 h, and the images were captured with a digital camera. The results were analyzed by ImageJ.

### 2.10 Colony Formation Assay

Colony formation was performed to evaluate the influence of different formulations on the recovery capacity of cancer cells. Briefly, MCF-7 or MDA-MB-231 cells were seeded into 6-well plates at a density of 3000 cells/well in 1.5 mL of culture media, followed by incubation for 24 h. The media were then replaced with FBS-free media containing CDDP–BEZ NPs, CDDP, BEZ, or the mixture of dual drugs with a CDDP concentration of 1 μg/mL. After incubation for 2 weeks, the cells in each well were washed with PBS three times and then fixed with 4% formaldehyde solution for 15 min. 0.5% crystal violet was used to stain the colonies for 15 min, after which excessive dye was removed by washing with PBS. Colonies in each well were then observed with a Nikon TE2000 inverted microscope (Nikon, Tokyo, Japan), and the images were captured with a digital camera.

### 2.11 Cell Migration and Invasion Assay

The migration and invasion properties of cancer cells were performed with the transwell assay in MCF-7 and MDA-MB-231 cells. For the evaluation of migration, the cells were seeded into the transwell inserts at a density of 5 × 10^4^ cells per well in 200 μL of FBS-free media containing different formulations with the concentration of CDDP at 3 μg/mL. 600 μL of culturing media containing 10% FBS were added in the lower chamber as an attractant. After incubation for 24 h, the migrating cells on the lower surface of the insert were fixed and stained with 0.5% crystal violet. The migrating cells were observed with an inverted microscope, and the images were captured with a digital camera. For the quantification analysis, 33% acetic acid was used to lyse the crystal violet, and the ODs at 570 nm were measured by a microplate reader to calculate the cell migration ratio.

The invasion assay was performed with transwell devices precoated with PBS-diluted Matrigel (20%, v/v). Cells were seeded into inserts at a density of 5 × 10^4^ cells per well and incubated overnight. The culturing media were then replaced with FBS-free media containing different formulations with a concentration of CDDP at 3 μg/mL. 600 μL of culturing media containing 10% FBS were added in the lower chamber as an attractant. The invasive cells on the lower surface of the insets were then fixed, stained, observed, and quantified according to the protocols in the migration assay.

### 2.12 Cellular Uptake of Pt

The accumulation of Pt in the cells was assessed in MCF-7 and MDA-MB-231 cells. Cells were seeded into 12-well plates at a density of 1.5 × 10^5^ cells per well, followed by incubation for 24 h. The cells were then treated with FBS-free media containing CDDP–BEZ NPs, CDDP, or the dual drug mixture with a CDDP concentration of 1 μg/mL. After further incubation for 1, 2, and 4 h, cells were collected and counted, whereafter the cells were digested with concentrated HCl overnight. The Pt accumulation in cells was measured by ICP-MS.

### 2.13 Immunofluorescence Analysis In Vitro

Immunofluorescence staining was performed on MCF-7 and MDA-MB-231 cells to explore the mechanisms involved in the enhanced antitumor efficacy of CDDP–BEZ NPs. Cells were seeded in 12-well plates at a density of 1.5 × 10^5^ cells per well and incubated for 24 h. For the γ-H2A.X assay, cells were treated with different formulations with the concentration of CDDP at 2 μg/mL for 12 h before immunofluorescence staining. For the cleaved caspase-9 and cleaved caspase-3 assays, cells were treated with different formulations with the concentration of CDDP at 3 μg/mL for 12 h before immunofluorescence staining. After drug administration, the cells were washed with PBS three times and then fixed with 4% paraformaldehyde for 15 min. The immunofluorescence staining was then performed on cells according to the manufacturer’s instructions. The images were taken with an inverted fluorescence microscope (Nikon, Japan).

### 2.14 Mitochondrial Depolarization Analysis

The dissipation of mitochondrial membrane potential (MMP) was assessed with the JC-1 fluorescent probe in MCF-7 and MDA-MB-231 cells after treatment with different formulations. Cells were seeded in a 96-well black plate with clear bottom at a density of 1 × 10^4^ cells per well and incubated for 24 h. After incubation with different formulations with the concentration of CDDP at 3 μg/mL for 12 h, the cells were processed with the JC-1 fluorescent probe according to the manufacturer’s instructions. Cells treated with FBS-free culturing media were measured as blank control, and cells treated with carbonylcyanide-*p*-trifluoromethoxyphenylhydrazone (FCCP) were studied as positive control. The fluorescence intensities at Ex. 535 ± 17.5 nm/Em. 590 ± 17.5 nm (JC-1 aggregate) and Ex. 475 ± 20 nm/Em. 530 ± 15 nm (JC-1 monomer) were read with a microplate reader, and the relative dissipation of MMP was calculated as the ratio between the JC-1 aggregate and the monomer. Each assay was repeated in triplicates.

### 2.15 Western Blotting Analysis

Western blotting analysis was performed on MDa-MB-231 cells to investigate the inhibitory activity of CDDP–BEZ NPs on the downstream proteins, phosphor-AKT (p-AKT) and phospho-S6 ribosomal protein (p-S6), in the PI3K/mTOR signaling pathway. MDA-MB-231 cells were seeded into a 6-well plate and incubated for 24 h before the assay. The cells were then treated with different formulations with the concentration of CDDP at 1 μg/mL for 9 h, after which the cells were lysed and harvested with a radioimmunoprecipitation assay (RIPA) buffer containing a kinase/phosphatase inhibitor cocktail. After centrifuging at 12,500 rpm for 5 min, the total protein extracts were obtained as the supernatant. 15 μg of protein extracts were mixed with loading buffer and loaded onto sodium dodecyl sulfate-polyacrylamide gel electrophoresis (SDS-PAGE), followed by stacking at 80 V for 30 min and separation at 100 V for 50 min. The protein bands were then transferred to a poly(vinylidene difluoride) (PVDF) membrane, which was blocked with 5% bovine serum albumin (BSA) for 1.5 h at room temperature. The membrane was incubated with primary antibody (p-AKT, p-S6, pan-AKT, and pan-S6, 1:500; GAPDH, β-tubulin, 1:1000, rabbit antihuman) at 4 °C overnight, followed by further incubation with horseradish peroxidase (HRP)-conjugated secondary antibody (1:2000, goat anti-rabbit) at room temperature for 2 h. The labeled bands were detected with electrogenerated chemiluminescence (ECL) according to the manufacturer’s instructions, and the images were captured and analyzed by a LI-COR Odessy Fc imaging system (Lincoln, NE).

### 2.16 Tumor Growth Inhibition on a Three-Dimensional (3D) Tumor Spheroid Model

The antitumor efficacy of different formulations was further investigated on a 3D tumor spheroid model established with MCF-7 cells. To form the tumor spheroids, cells were seeded into an agarose-coated 96-well plate at a density of 5 × 10^4^ cells per well in 200 μL of complete media containing 0.25% (v/v) Matrigel Matrix. After centrifugation at 1200 rpm for 10 min, the cells were incubated for 2 weeks for the formation of tumor spheroids. The as-established tumor spheroids were then transferred to a 48-well plate and treated with different formulations with the concentration of cisplatin at 5 μg/mL. The growth of tumor spheroids was observed for a week with an inverted microscope, and the images were captured with a digital camera on days 1, 3, 5, and 7. The size of the tumor spheroids was analyzed with ImageJ. On day 7, a live/dead staining assay was performed on tumor spheroids. The images were taken with an inverted fluorescence microscope (Nikon, Japan).

### 2.17 In Vivo Tumor Growth Inhibition Study

All of the animal experiments were performed under the approval of the Guidelines for the Care and Use of Laboratory Animals of Central South University. Six-week-old male BALB/c mice were purchased from Hunan SJA Laboratory Animal Co., Ltd. The in vivo experiments were conducted on a 4T1 subcutaneous tumor model, which was established by subcutaneous injection of 1 × 10^7^ 4T1 cells suspended in 200 μL of sterile PBS into the right flank of mice. For the in vivo tumor growth inhibition study, mice were randomly divided into seven groups (six mice/group), which were treated as follows: (1) saline, (2) CDDP–BEZ NPs, (3) physical mixture of BEZ and CDDP (BEZ solution mixed with CDDP solution), (4) BEZ, (5) CDDP, (6) 10 ng/mL insulin and CDDP–BEZ NPs, and (7) 10 ng/mL insulin and the physical mixture of BEZ and CDDP. All the treatments were administrated to mice via intratumoral injection of 100 μL of PBS comprising therapeutic agents equivalent to CDDP–BEZ NPs containing 10 μg/mL CDDP. Treatments were performed every 2 days for 12 days in total. The tumor volume and body weight of the mice were recorded. All mice were humanely sacrificed after the last record to obtain their tumors, which were preserved in 4% paraformaldehyde fixative. All tissues were then sent to Wuhan Sevier Biological Co., Ltd. for further analysis. The tissues were preserved in a 10% formalin solution, embedded in paraffin, sliced into sections, and stained using various antibody-dye combinations. Afterward, all samples underwent hematoxylin and eosin (H&E) staining followed by imaging.

### 2.18 Statistical Analysis

Statistical analysis was performed with Origin2021 (Origin lab). Experimental data were presented as means ± standard deviation. Values were compared with one-way analysis of variance (ANOVA) analysis. A *p*-value less than 0.05 was considered significantly different.

## 3 Results and Discussion

### 3.1 Preparation and Characterization of CDDP–BEZ NPs

The high hydrophobicity of BEZ hinders its clinical translation, which might be addressed by modification with a hydrophilic ligand or encapsulation into nanoDDS. Inspired by the application of a platinum linker in improving the aqueous solubility of BEZ, the hydrophilic CDDP was conjugated with BEZ through a coordination bond between Pt and the N atom on the quinoline group in the imidazoquinoline of BEZ.^17^ The successful coordination not only improved the aqueous solubility of BEZ but also led to the formation of self-assembled NPs due to the hydrophobicity of BEZ and the high polarity of CDDP.

**Figure 1:**
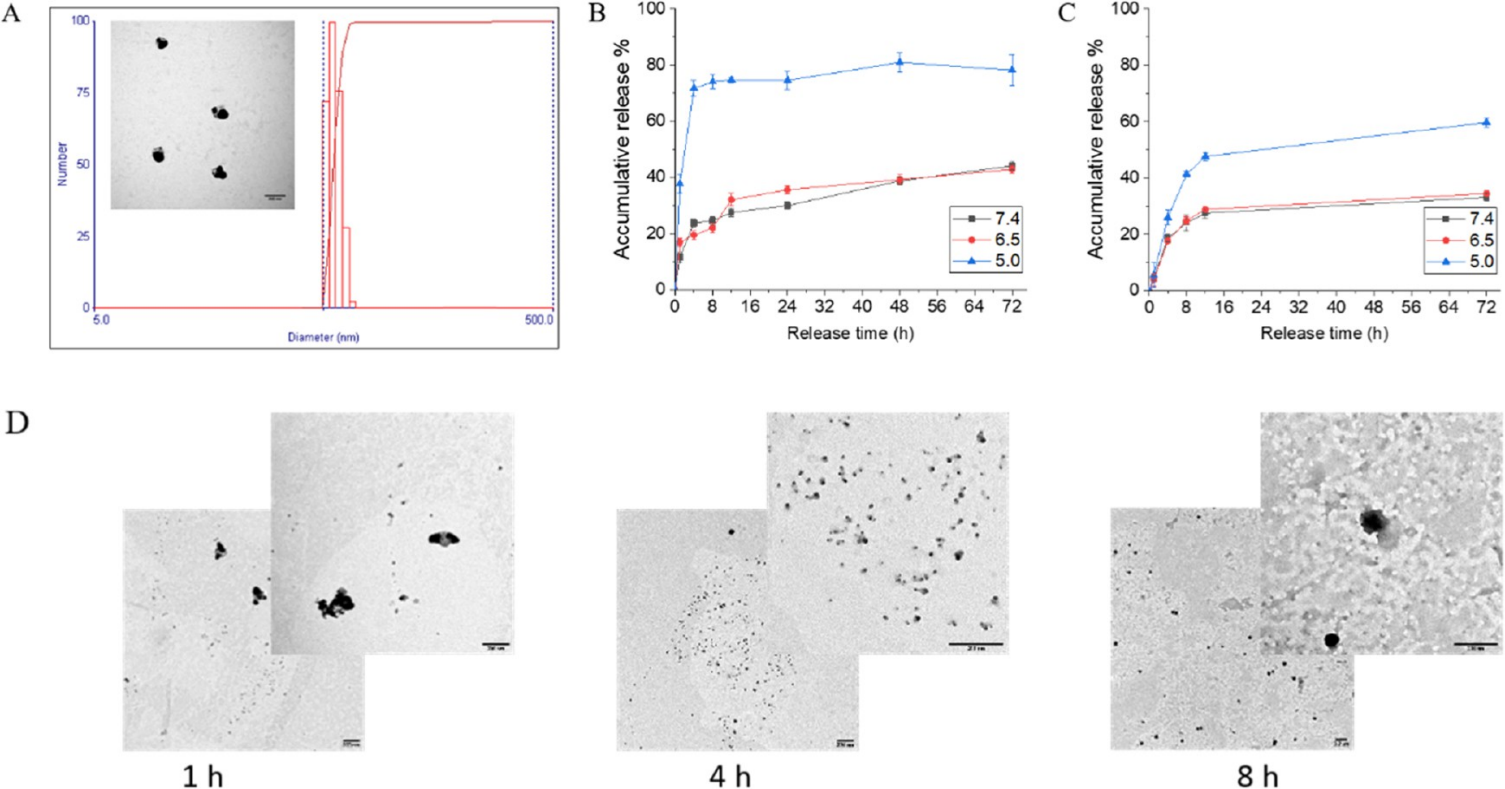
Physicochemical characterization of CDDP–BEZ NPs. (A) Particle size of CDDP–BEZ NPs analyzed by DLS; the inserted picture was the particle morphology observed with TEM. (B) pH-responsive drug release of BEZ from CDDP–BEZ NPs. (C) pH-responsive drug release of CDDP from CDDP–BEZ NPs. (D) Disintegration of CDDP–BEZ NPs after incubation in PBS at pH 5.0 for different time durations (scale bar = 200 nm).

Despite the difficulties with controllable size and uniformity of carrier-free nanoDDS reported in previous studies, CDDP–BEZ NPs exhibited uniform size and morphology without the assistance of any templates.^15,18^ As demonstrated in Figure 1A, CDDP–BEZ NPs were uniform quasispheres with an average hydrodynamic diameter of 136.5 ± 0.7 nm and a PDI of 0.182. With a positive ζ potential of +39.25 ± 5.25 mV, CDDP–BEZ NPs exhibited good stability, and their particle size remained nearly unchanged during storage for 2 months at 5 °C in the dark (Figure S1).

### 3.2 pH-Responsive Disintegration and Drug Release Profile of CDDP–BEZ NPs

Due to the susceptibility of the Pt-based coordination bond, CDDP–BEZ NPs exhibited a pH-responsive drug release profile.^19^ Incubation of CDDP–BEZ NPs in an acidic environment (PBS at pH 5.0) led to a burst release of BEZ (71.75%) within the first 4 h and a drug release ratio up to 80.98% after a 72 h incubation period, while for the NPs incubated in a neutral (PBS at pH 7.4) or slightly acidic (PBS at pH 6.5) environment, the final release after 72 h was only approximately 40% (Figure 1B). Poor stability has remained a challenge to be addressed in the design of a carrier-free drug delivery system.^20^ However, our results suggested that CDDP–BEZ NPs could maintain their morphology in neutral or slightly acidic PBS (Figure S2). By contrast, a fast disassembly of NPs was induced by PBS at pH 5.0, which was consistent with the pH-responsive drug release profile. As shown in Figure 1C, the disassociation of CDDP–BEZ NPs started after incubation for 1 h, and most CDDP–BEZ NPs disappeared after incubation for 8 h. It is well-elucidated that the tumor microenvironment is acidic, and therefore, the sensitive pH-responsiveness due to the Pt-based coordination bond made CDDP–BEZ NPs an effective and safe delivery strategy for antitumor therapy, by which a quick kill of tumor tissues could be achieved without harming the adjacent normal tissues.^19,21^

### 3.3 In Vitro Cytotoxicity Study on Cancer Cells

**Figure 2:**
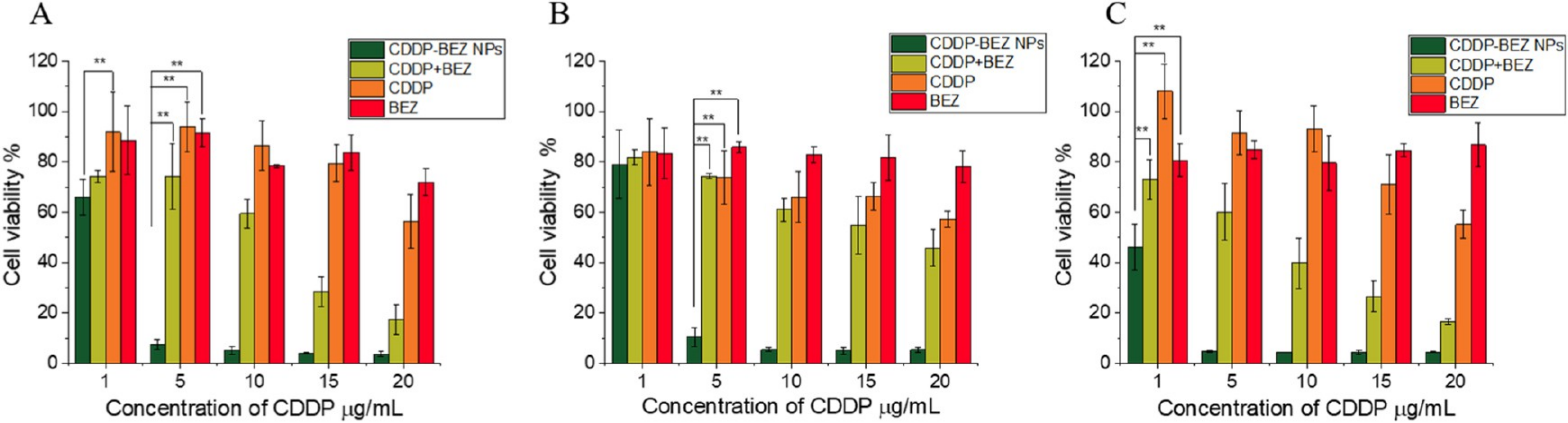
In vitro anticancer efficacy study. In vitro cell cytotoxicity on (A) human breast cancer cell lines MCF-7, (B) MDA-MB-231, and (C) human ovarian cancer cell line OVCAR-3 after incubation with different formulations for 24 h. **, *p* < 0.05.

The in vitro cytotoxicity of CDDP–BEZ NPs was investigated in cancer cell lines MCF-7, MDA-MB-231, and OVCAR-3 by an MTT assay. CDDP–BEZ NPs exerted an obvious enhancement in the inhibitory effect on the proliferation of all tested cell lines (Figures 2 and S3). Compared with free drugs, the IC_50_ values of both CDDP and BEZ were greatly reduced by CDDP–BEZ NPs (Table S1), suggesting promoted cytotoxicity. In addition, a lower combination index (CI) value (Table S2) at all levels further indicated that a facilitated synergistic efficacy between CDDP and BEZ could be achieved through the codelivery system in comparison with the physical mixture of dual drugs. The superiority of CDDP–BEZ NPs over the dual drug mixture in combating the proliferation of cancer cells might be attributed to the simultaneous entrance of both drugs into cancer cells, which might be further amplified by the sequential and precise drug release due to the pH-responsiveness of the NPs.^22^ Therefore, CDDP–BEZ NPs showed highly effective cytotoxicity in both healthy (Figure S4 and Table S1) and cancer cell lines, using much lower therapeutic doses than those required when using CDDP and BEZ separately or mixed.

**Figure 3:**
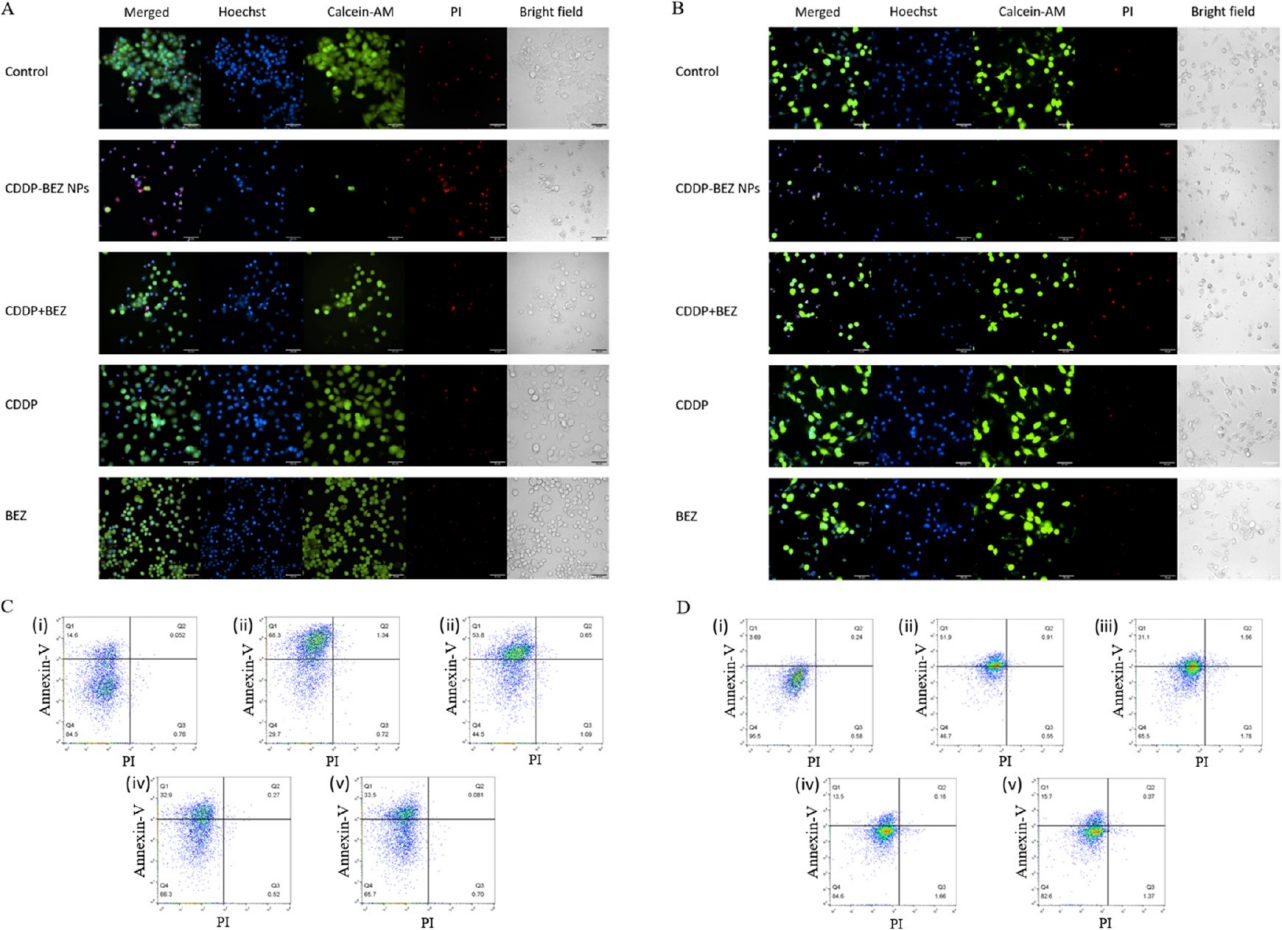
In vitro cell cytotoxicity investigated by live and dead staining of (A) MCF-7 and (B) MDA-MB-231 cells. Annexin V/PI double-staining flow cytometry analysis of cell apoptosis (C) MCF-7 and (D) MDA-MB-231 cells: (i) control group, (ii) cells treated with CDDP–BEZ NPs, (iii) cells treated with the mixture of BEZ and CDDP, (iv) cells treated with CDDP, and (v) cells treated with BEZ. *Y*-axis: Annexin V and *X*-axis: PI. Q4: living cells. Upper panel (Q1, Q2): apoptotic cells.

The results of live and dead staining suggested that at the same dosage, treatment in both tested cancer cell lines with CDDP–BEZ NPs led to more cell death compared with other formulations (Figure 3A,B). Noteworthy, shrinkage of the nucleus was observed in most cells treated with CDDP–BEZ NPs, suggesting the initiation of apoptosis with this treatment.^23^ This finding was in line with the result of a flow cytometry study, where more obvious apoptosis was induced in MCF-7 (68.3%) and MDA-MB-231 cells (51.9%) by CDDP–BEZ NPs compared with either free drugs or the dual drug mixture at equivalent treatment dosage (Figure 3C,D).

Hyperglycemia (high blood glucose) is one major on-target toxicity of PI3K/AKT inhibitors to systematic metabolism, consequently leading to hyperinsulinemia (high blood insulin).^24^ The excessive insulin would then bind with insulin receptor (IR) or insulin growth factor 1 (IGF-1) and reactivate the PI3K signaling axis in return, compromising the therapeutic efficacy of PI3K/AKT inhibitors.^25^ To explore if the codelivery with CDDP could combat the above-mentioned negative feedback, the cytotoxicity of CDDP–BEZ NPs was further evaluated with the presence of insulin. A noticeable elevation in the cytotoxicity of CDDP-containing formulations was observed in all of the investigated cell lines (Figure S5). Insulin in combination with CDDP was reported to facilitate the apoptosis in ovarian cancer cells through the activation of p53 and the JNK signaling pathway, which may explain the augmentation in cytotoxicity in this study.^26^ Therefore, the codelivery of CDDP and BEZ could not only explicitly promote cytotoxicity in cancer cell lines but also maintain the capacity to reverse the potential negative feedback from insulin.

### 3.4 Impairment in Recovery, Migration, and Invasiveness in Cancer Cells Induced by CDDP–BEZ NPs

**Figure 4:**
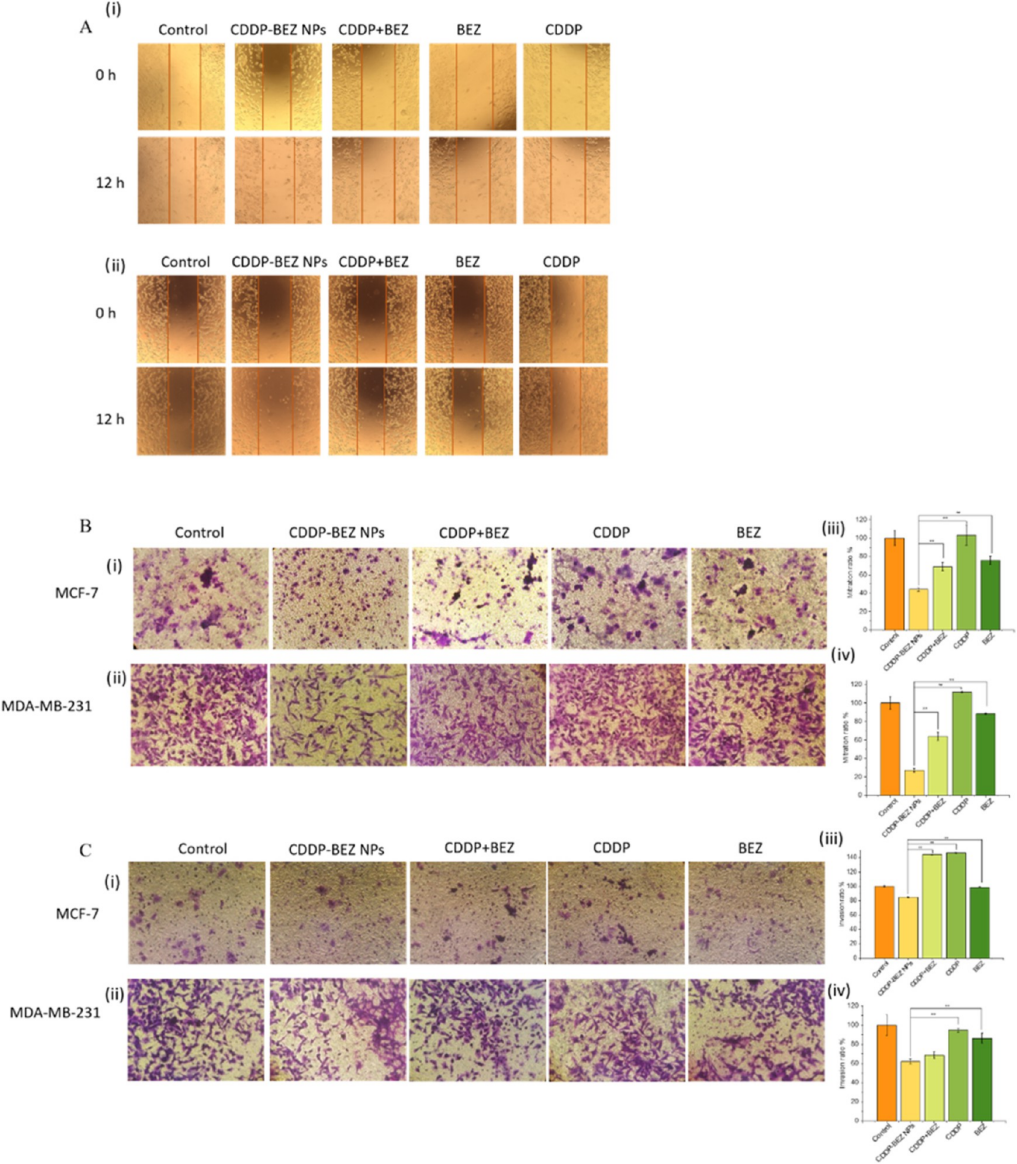
Influence on the migration and invasion properties of cancer cells induced by different formulations. (A) Wound healing assay on (i) MCF-7 and (ii) MDA-MB-231 cells; (B) transwell migration assay on (i) MCF-7 and (ii) MDA-MB-231 cells and the respectively quantitative analysis (iii, iv); (C) invasion assay on (i) MCF-7 and (ii) MDA-MB-231 cells and the respective quantitative analysis (iii, iv). **, *p* < 0.05.

Apart from the strong antiproliferative effect, it was found that CDDP–BEZ NPs manifested excellent antimetastasis properties, exhibiting potent hindrance in the recovery, migrating, and invasive capacity of cancer cells. A scratch assay was first performed to investigate the influence of different formulations on cell recovery ability. As shown in Figure 4A, only CDDP–BEZ NPs aggravated the scratch in the cell monolayer, while the cells treated with other formulations resulted in scratch closure to different extents. The impairment in the recovery from treatment in cancer cells was then validated by a colony formation assay, with no trace of colony formation shown in cells treated with CDDP–BEZ NPs (Figure S6). The impact of different treatments on cancer cell migration was evaluated by a transwell assay, where the least chamber crossing was induced by the administration of CDDP–BEZ NPs (Figure 4B). Consistently, the results of the invasion assay demonstrated that CDDP–BEZ NPs could effectively prevent cancer cells from penetrating the extracellular matrix (Figure 4C). Monotherapy with CDDP has been reported to induce metastasis in various cancers, which was also presented in our findings with augmented migrating ability observed in the cells treated with CDDP alone.^27^ However, coadministration of CDDP with BEZ could attenuate the CDDP-induced metastasis as the result of interference on the PI3K/mTOR signaling pathway.^28^ Besides, it should be noted that a more obvious antimetastatic effect from CDDP–BEZ NPs was observed in MDA-MB-231 cells in comparison with MCF-7 cells, suggesting that CDDP–BEZ NPs held the potential for the treatment of cancers with high malignancy and invasiveness.^29^

### 3.5 CDDP–BEZ NPs Augmented the Damage to DNA

**Figure 5:**
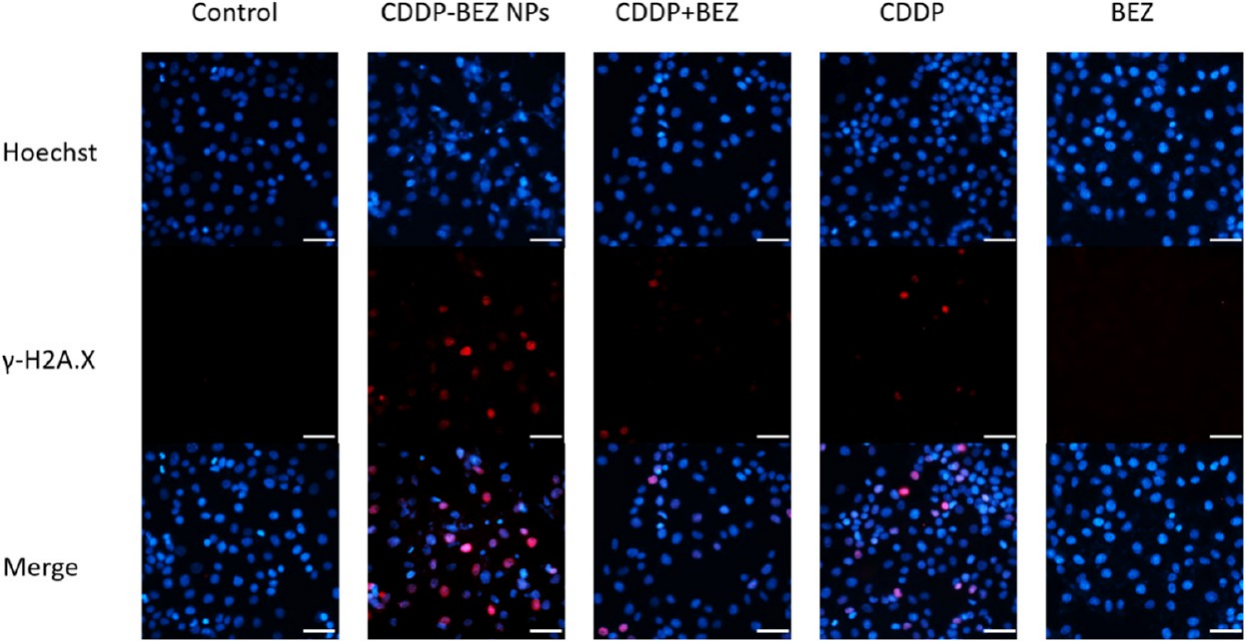
Immunofluorescence of γ-H2A.X in MCF-7 cells treated with different formulations (scale bar = 50 μm).

The superiority of nanoDDS over free CDDP in facilitating the cellular uptake of Pt has been widely elucidated in previous studies, which is in line with our study.^30,31^ We hypothesized that CDDP–BEZ NPs would rapidly disintegrate and release CDDP due to its sensitive pH-responsiveness and that the released CDDP would subsequently form an adduct with DNA, causing DSB and killing cancer cells as a result. This hypothesis was confirmed by examining DNA damage via the immunofluorescence staining of γ-H2A.X, a phosphorylated histone protein accumulating on DNA damage sites immediately after DNA breaks.^32^ The highest level of γ-H2A.X was observed in cells treated with CDDP–BEZ NPs (Figures 5 and S7), suggesting that the most severe DNA damage was induced by the codelivery of CDDP and BEZ because of the elevated accumulation and rapid release of Pt.

### 3.6 CDDP–BEZ NPs Induced the Disruption of Mitochondria

**Figure 6:**
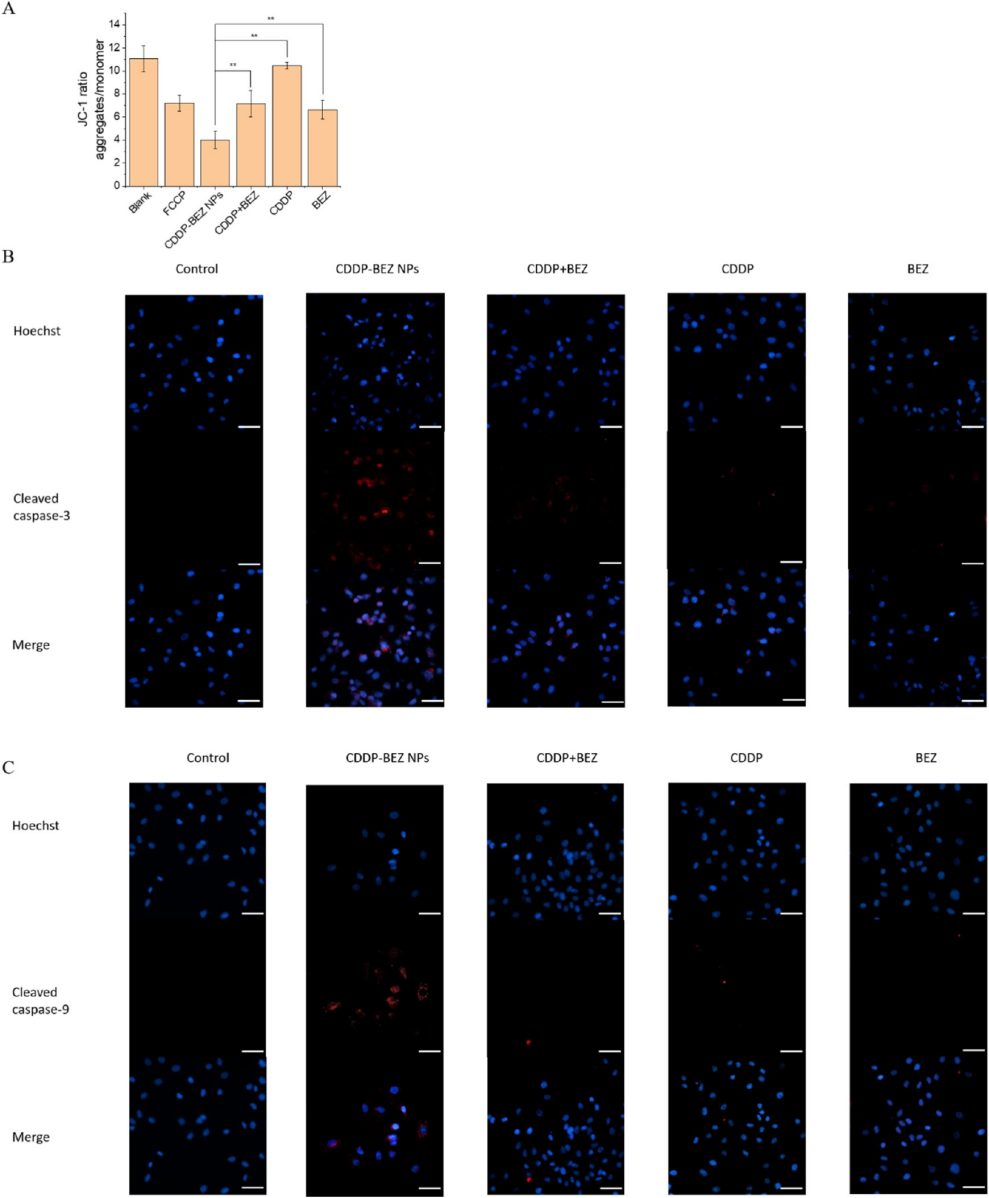
CDDP–BEZ NPs activated the intrinsic apoptosis pathway by disrupting mitochondria in MCF-7 cells. (A) Mitochondrial potential measured by the JC-1 assay in MCF-7 cells treated with different formulations. (B) Immunofluorescence of cleaved caspase-3 and (C) cleaved caspase-9 in MCF-7 cells treated with different formulations (scale bar = 50 μm). **, *p* < 0.05.

Nucleotide excision repair (NER) has been reported as a major mechanism in repairing CDDP-DNA adducts, contributing to the development of resistance to CDDP.^4^ Inspired by the lack of NER in mitochondrial DNA, various strategies have been designed to deliver CDDP to mitochondria.^33,34^ Results of these studies suggested that well-regulated lipophilicity with suitable modification could increase the mitochondrial accumulation of CDDP.^25,35^ Therefore, we hypothesized that the coordinated CDDP–BEZ dissociated from the NPs may also affect mitochondria. Mitochondrial membrane potential (MMP, ΔΨ_m_) was first assessed with a JC-1 fluorescent probe to evaluate the impact of different formulations on the structure of mitochondria. As demonstrated in Figures 6A and S10, compared with CDDP alone, the introduction of BEZ contributed to a more evident MMP dissipation, which was further exacerbated by CDDP–BEZ NPs. The aggravated mitochondrial depolarization in cells treated with both CDDP and BEZ could be attributed to the inhibition of the PI3K/mTOR signaling pathway, where elevated mitochondrial cytotoxicity was the consequence of the dysfunctional mitochondria–lysosome crosstalk.^9^ The disruption of mitochondria led to the activation of caspases, further contributing to the apoptosis of cancer cells.^36^ Consistent with their influence on MMP, CDDP–BEZ NPs resulted in intense activation of caspase-9 and caspase-3, with prominent fluorescence observed after immunofluorescence staining (Figures 6B,C and S8). In comparison, cells treated with the mixture of dual drugs showed only mild increases in caspase-3 but not caspase-9. Collectively, our findings suggested that the induction of mitochondria-dependent apoptosis contributed to the boosted antitumor efficacy of CDDP–BEZ NPs.

### 3.7 Western Blotting Analysis

As a PI3K/mTOR dual inhibitor, BEZ exerts its antitumor function by interfering with the phosphorylation process of this signaling axis.^37^ Therefore, the phosphorylation level of AKT and S6 ribosomal was analyzed to evaluate if CDDP–BEZ NPs still maintained kinase inhibitory function. Results of Western blotting analysis (Figure S9) suggested that treatment with CDDP–BEZ NPs reduced the phosphorylation of both AKT (p-AKT) and S6 (p-S6) to a greater extent compared with other BEZ-containing formulations at investigated conditions. It should be noted that the suppression of the phosphorylation level on p-S6 was more obvious, which might be attributed to the triple inhibition on p-AKT, mTOR2, and mTOR1. Similar results have been elucidated in a previous study, where the phosphorylation inhibitory activity of BEZ at a low dose (<50 nM) was greatly boosted by a suitable nanodelivery system, as the benefits of the promoted cellular uptake and sustained drug release profile.^38^ One major hindrance in the clinical translation of BEZ was its unfavorable toxicity profile led by the high administration dose.^39^ Therefore, by elevating the kinase inhibitory function of BEZ at low doses, CDDP–BEZ NPs might serve as a promising strategy to improve its clinical perspectives.

### 3.8 CDDP–BEZ NPs Suppressed the Growth and the Metastasis on 3D Tumor Spheroids

**Figure 7:**
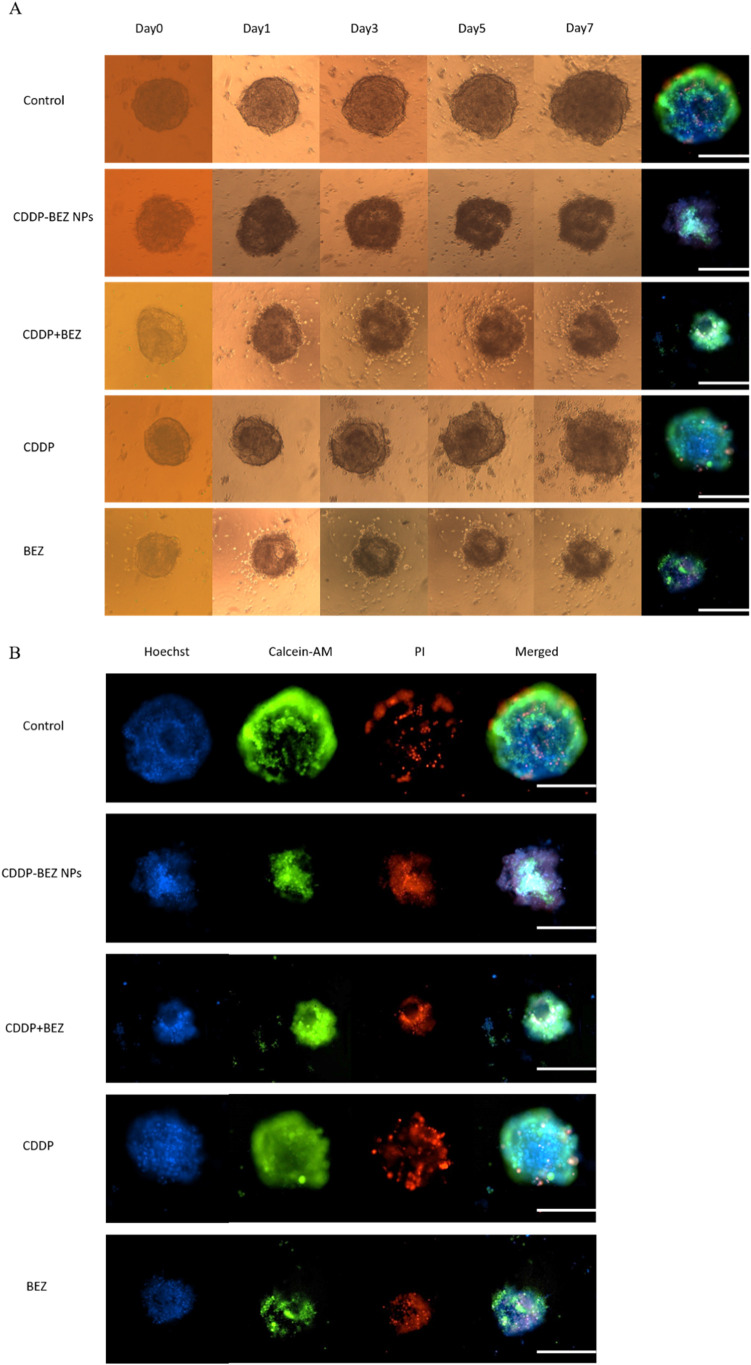
Growth inhibition study on 3D tumor spheroids. (A) Growth inhibition on single cell line tumor spheroids of MCF-7. (B) Live/dead staining on tumor spheroids at the end of the treatment (scale bar = 250 μm).

Given that the interaction between the tumor and the tumor microenvironment presents multiple barriers against drug penetration, accumulation, and efficacy, the conventional 2D cell cultures may not be enough to reflect the actual antitumor behavior of nanoDDS.^40^ To evaluate if CDDP–BEZ NPs could still manifest their superior antitumor efficacy under a complicated environment, the growth inhibition on 3D tumor spheroids was continuously monitored for 1 week (Figure 7A). At the investigated dose, CDDP alone failed to constrain the growth of tumor spheroids and initiated the migration of cancer cells from the original spheroid. Though BEZ alone or in combination with CDDP could inhibit the growth of the tumor spheroid, migration of massive live tumor cells was observed (Figure 7B), suggesting the possibility of inducing metastasis with this therapy. By contrast, CDDP–BEZ NPs not only effectively limited the growth of tumor spheroids but also restrained the potential metastasis, with no sign of cell migrating observed during the whole treatment. It has been reported that some miRNAs participated in the CDDP-induced metastasis through the activation of the PI3K signaling pathway.^41^ Therefore, the codelivery of CDDP and BEZ could suppress the metastasis by combating the dysfunction of the PI3K signaling axis. Besides, the elevation in the kinase inhibitory activity of BEZ by CDDP–BEZ NPs at the investigated dose may also contribute to superior therapeutic outcomes.^38^ The potent inhibition on the growth and metastasis of tumor spheroids by CDDP–BEZ NPs suggested that the as-prepared NPs could maintain the superior antitumor efficacy even under a complicated microenvironment, as the result of multiple mechanisms, including the augmented DNA damage, the activated mitochondria-dependent apoptosis, and the facilitated inhibition on the PI3K/mTOR signaling pathway. Therefore, it is reasonable to conclude that CDDP–BEZ NPs maintain the perspective of acting as a functional therapeutic platform for the treatment of solid tumors.

### 3.9 CDDP–BEZ NPs Impaired the Development of Tumor in Mice

**Figure 8:**
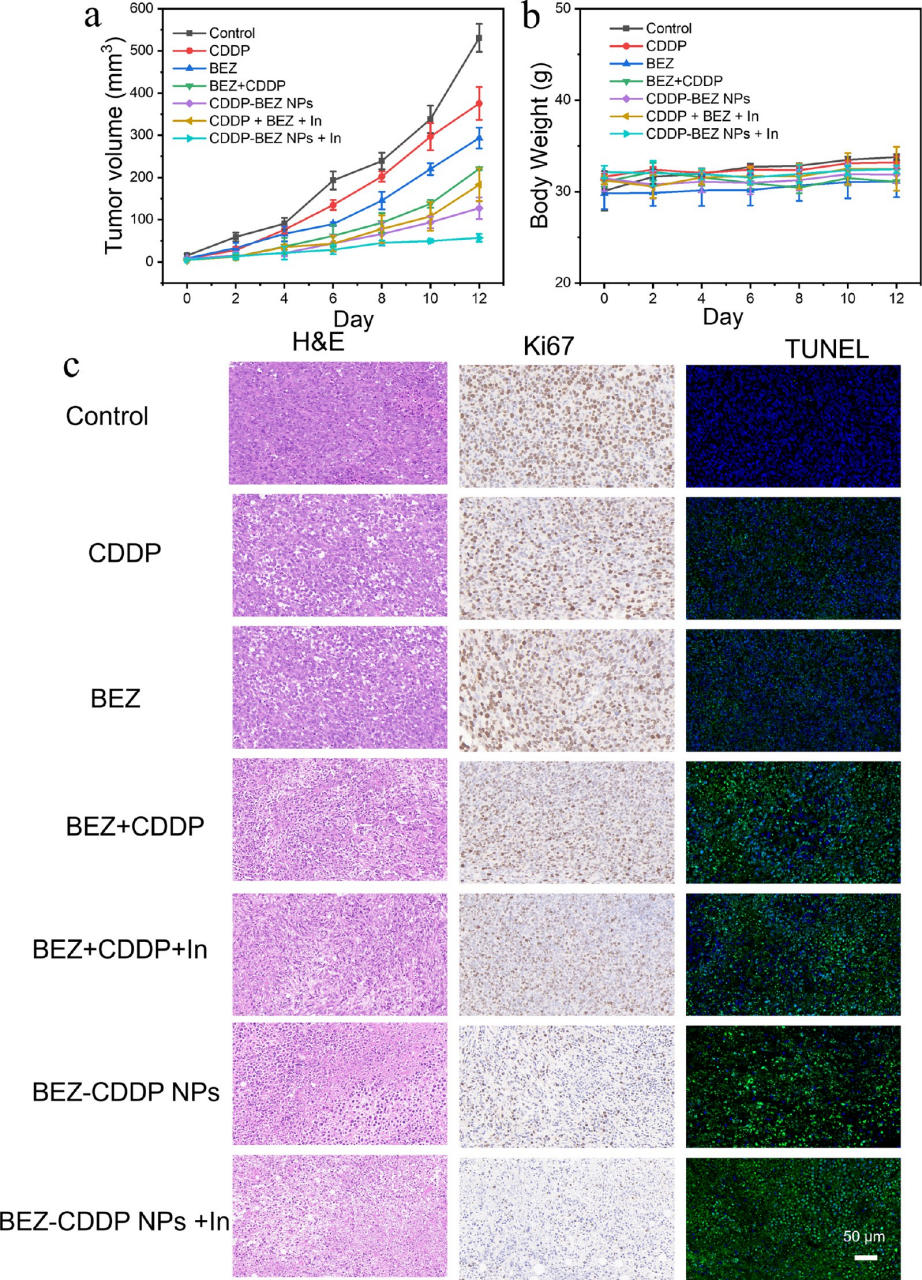
In vivo antitumor effects of CDDP–BEZ NPs. (A) Tumor volume growth curves after treatment with saline, CDDP, BEZ, BEZ + CDDP, CDDP–BEZ NPs, CDDP + BEZ + insulin, and CDDP–BEZ NPs + insulin. The volume of the tumor was measured after each administration. (B) Body weight of the tested mice during the whole treatment period. (C) Representative hematoxylin and eosin (H&E), Ki67, and TUNEL staining in continuous sections from tumors in different treating groups. The scale bar indicates 50 μm.

Consistent with the results of the in vitro study, CDDP–BEZ NPs-treated groups exhibited the most prominent antitumor efficacy in the in vivo study (Figure 8A), where remarkably slower tumor growth of the mice administrated with both CDDP–BEZ NPs and insulin was observed, further demonstrating the feasibility of combating the negative insulin feedback on most PI3K/AKT inhibitors through the codelivery of CDDP. Noteworthily, the administration of CDDP–BEZ NPs, with or without the addition of insulin, led to the drastic reduction of proliferating cells in tumors compared with other treatment groups, as suggested by the results of Ki67 staining in Figure 8C. This finding was in line with the result of the in vitro colony formation assay (Figure S6), validating that CDDP–BEZ NPs could prevent cancer cells from recovering after treatment. By contrast, monotherapy with either CDDP or BEZ exerted poor antitumor efficacy, with limited inhibition on tumor size, little apoptosis in tumor tissue, and a massive amount of proliferating cells, which justified the potency of combining CDDP and BEZ for a better antitumor efficacy, as suggested by previous study.^10^ However, a major amount of proliferating cells could be observed in tumor slices from mice treated with the physical mixture of CDDP and BEZ after Ki67 staining, despite the inhibition of tumor volume by the treatment with a dual drug mixture, emphasizing the prominence of a suitable delivery system for both drugs for the ideal therapeutic outcomes.^7^ In addition, no obvious change in body weight was observed in the tested mice during the whole treatment period (Figure 8B), reflecting the encouraging safety profile of CDDP–BEZ NPs. Therefore, CDDP–BEZ NPs have been validated to hold the potential to suppress tumor progress with high efficacy, promising safety, and inspiring prognosis. Taken together, both in vivo and in vitro experiments have shown that CDDP–BEZ NPs might lead to less adverse reactions, offering a safer therapeutic strategy than the mixed administration of dual drugs. It is worth noting that the highly positive ζ potential of CDDP–BEZ nanoparticles (NPs) without a protecting layer is likely to promote significant protein adsorption from biological environments, leading to the formation of a protein corona. This protein corona can dramatically alter the surface properties of the nanoparticles, influencing their behavior in both in vitro and in vivo conditions. For instance, it may affect cellular uptake, biodistribution, immune recognition, and overall therapeutic efficacy. In in vitro systems, this may result in altered nanoparticle–cell interactions, while in vivo, the protein corona could change pharmacokinetics, potentially reducing circulation time and increasing clearance by the reticuloendothelial system. This will require systematic future studies.

## 4 Conclusions

In this study, we proposed an innovative pure drug nanoDDS, CDDP–BEZ NPs, for the codelivery of two anticancer drugs with distinguished differences in physicochemical properties. Through the self-assembly of the coordinated conjugate CDDP–BEZ, this system achieved the self-delivery of two chemotherapeutic agents, promoting synergistic therapeutic efficacy while eliminating the potential side effects from the vector material. Facilitated cellular accumulation and sensitive pH-responsiveness of CDDP–BEZ NPs boosted the synergy in the inhibition of the development and progression of cancers both in vitro and in vivo, leading to the escalation in antiproliferative efficacy, the deterioration in the recovery ability, and the impairment in migration and invasion properties in cancer cells. The mechanism study revealed that the increase in DNA damage, the interference of the phosphorylation process in the PI3K signaling pathway, and the initiation of mitochondria-dependent apoptosis all participated in augmenting the antitumor synergy of CDDP–BEZ NPs. Driven by the collaboration of multiple mechanisms, CDDP–BEZ NPs maintained superior excellence in combating tumor progression even under a complicated physiological environment. Collectively, we believe that CDDP–BEZ NPs presented a carrier-free strategy to address the existing hindrance in the combination therapy of Pt-based chemo agents and PI3K/mTOR dual inhibitors, which holds a perspective for further industrial and clinical applications, offering a possibility for enhanced therapeutic outcomes in treating solid tumors.

## Supporting Information Available

Particle size of CDDP–BEZ NPs measured by DLS for 2 months (Figure S1); morphology of CDDP–BEZ NPs after incubation with PBS at pH 7.4 or 6.5 for different time durations (Figure S2); in vitro anticancer efficacy study for 72 h (Figure S3); in vitro cytotoxicity on normal tissue cells (Figure S4); in vitro cytotoxicity with the existence of insulin (Figure S5); colony formation assay (Figure S6); immunofluorescence of γ-H2A.X in MDA-MB-231 cells treated with different formulations (Figure S7); CDDP–BEZ NPs activated the intrinsic apoptosis pathway by contributing to mitochondrial depolarization in MDA-MB-231 cells (Figure S8); inhibition on the phosphorylation process of the PI3K signaling pathway (Figure S9); IC_50_ value of CDDP in different formulations (Table S1-1); IC_50_ value of BEZ in different formulations (Table S1-2); CI value in MCF-7 cells (Table S2-1); CI value in MDA-MB-231 cells (Table S2-2); and CI value in OVCAR-3 cells (Table S2-3) (PDF)
