# Supplementary material for: Carrier-Free Cisplatin–Dactolisib Nanoparticles for Enhanced Synergistic Antitumor Efficacy
Source: ACS Biomater Sci Eng. 2025 Feb 24;11(3):1456–71. doi: 10.1021/acsbiomaterials.4c00672 (PMC11897951; doi:10.1021/acsbiomaterials.4c00672)
Supplement: Supplementary file 1 — ab4c00672_si_001.pdf [file ab4c00672_si_001.pdf]

Supporting Information

Carrier-free cisplatin-dactolisib nanoparticles for enhanced synergistic anti-tumor efficacy

Mei Zhang[a, b], Qiuxia Tan[c], Sevil Gonca[a], Minhuan Lan[c], Bin-Zhi Qian[d], Xianfeng Chen[a*], Norbert Radacsi[a]*

Pages: 11 pages

Number of figures: 9

Number of tables: 5

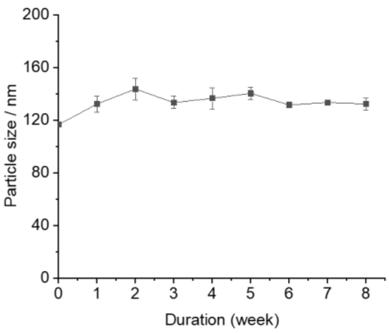

Figure S1. Particle size of CDDP-BEZ NPs measured by DLS for 2 months (storage at 5°C in the dark).

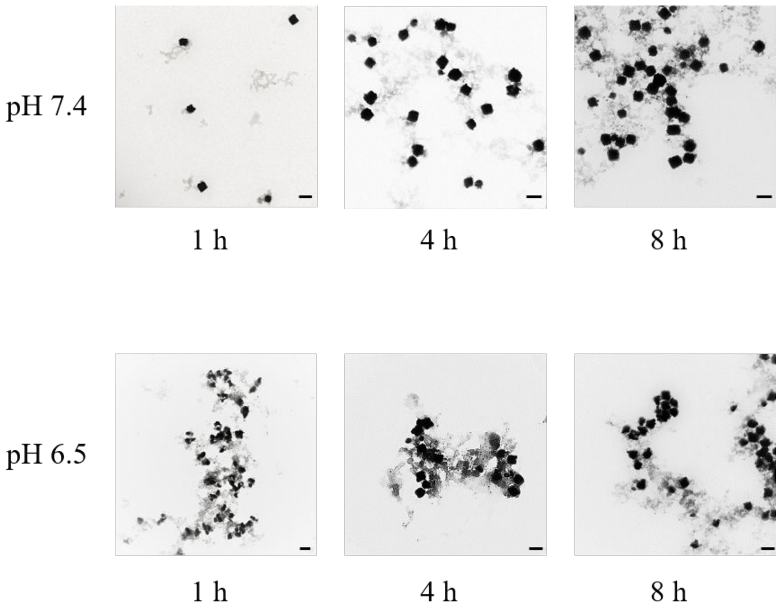

Figure S2. Morphology of CDDP-BEZ NPs after incubation with PBS at pH 7.4 or 6.5 for different time duration (Scale bar = 200 nm).

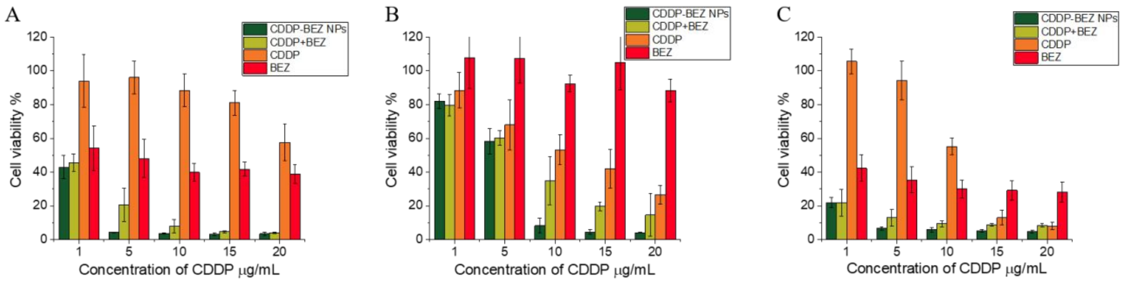

Figure S3. In vitro anti-cancer efficacy study for 72 h. In vitro cell cytotoxicity on (A) human breast cancer cell lines MCF-7, (B) MDA-MB-231, and (C) human ovarian cancer cell line OVCAR-3 after incubation with different formulations for 72 h.

Table S1-1. IC_{50} value of CDDP in different formulations (μg/mL)

	MCF-7	MDA-MB-231	OVCAR-3	MCF-10A	HUVEC
CDDP-BEZ NPs	1.30	1.94	0.55	1.13	1.48
Dual drug mixture	6.92	21.52	4.63	3.31	1.18
CDDP	130.48	56.96	119.18	9.82	4.30

Table S1-2. IC_{50} value of BEZ in different formulations (μg/mL)

	MCF-7	MDA-MB-231	OVCAR-3	MCF-10A	HUVEC
CDDP-BEZ NPs	2.02	3.02	0.86	1.75	2.31
Dual drug mixture	4.88	2.41	7.22	5.17	1.84
BEZ	169.92	160.94	134.57	6.39	0.60

Table S2-1. CI value in MCF-7 cells

	**ED_{50}**	**ED_{75}**	**ED_{90}**	**ED_{95}**
CDDP-BEZ NPs	0.13366	0.15032	0.22397	0.29444
Dual drug mixture	0.21944	0.44128	1.17569	2.29502

Table S2-2. CI value in MDA-MB-231 cells

	**ED_{50}**	**ED_{75}**	**ED_{90}**	**ED_{95}**
CDDP-BEZ NPs	0.01331	0.00637	0.00308	0.00188
Dual drug mixture	0.31103	0.35506	0.40967	0.45184

Table S2-3. CI value in OVCAR-3 cells

	**ED_{50}**	**ED_{75}**	**ED_{90}**	**ED_{95}**
CDDP-BEZ NPs	0.03614	0.03124	0.03344	0.0354
Dual drug mixture	0.26556	0.3887	0.70461	1.06735

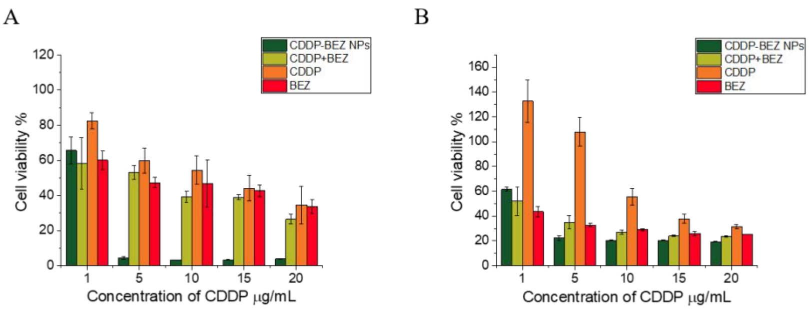

Figure S4. *In vitro* cytotoxicity on normal tissue cells. In vitro cell cytotoxicity on (A) human breast cell line MCF-10A, and (B) human umbilical vein endothelial cell line HUVEC after incubation for 24 h.

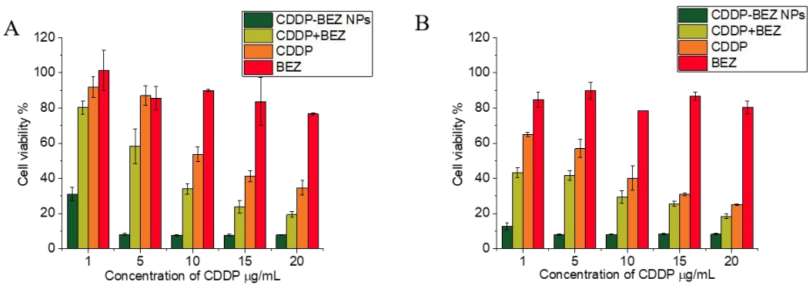

Figure S5. *In vitro* cytotoxicity with the existence of insulin. In vitro cell cytotoxicity on (A) MCF-7, and (B) MDA-MB-231 with the existence of insulin (10 ng/mL).

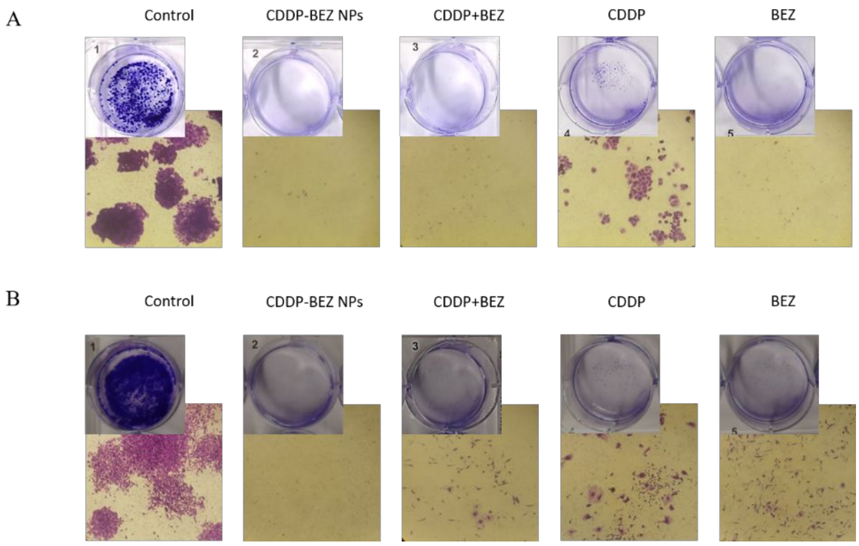

Figure S6. Colony formation assay. Colony formation after (A) MCF-7 and (B) MDA-MB-231 cells were treated with different formulations for 24 h before incubation for 2 weeks.

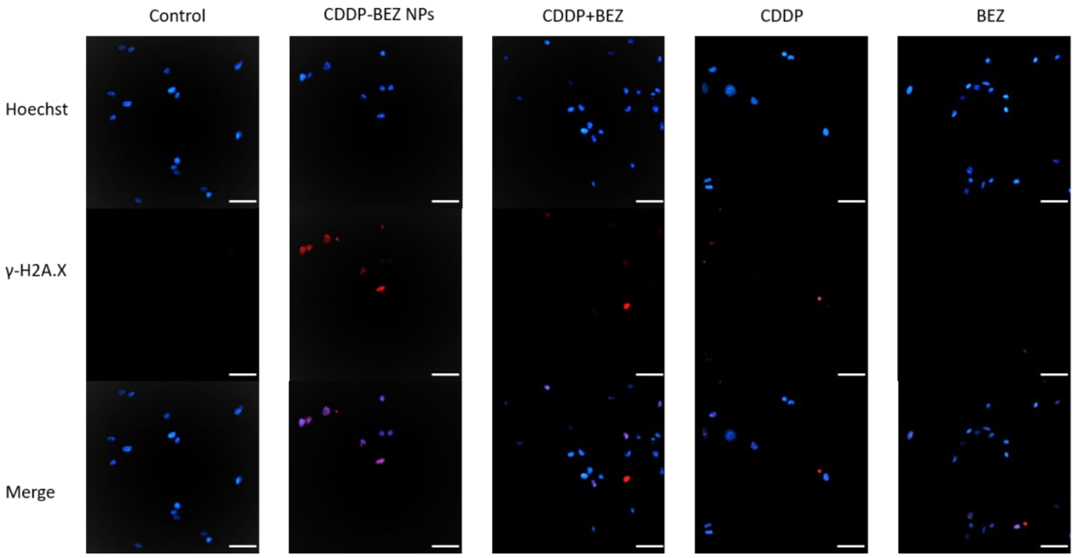

Figure S7. Immunofluorescence of γ-H2A.X in MDA-MB-231 cells treated with different formulations (Scale bar = 50 μm).

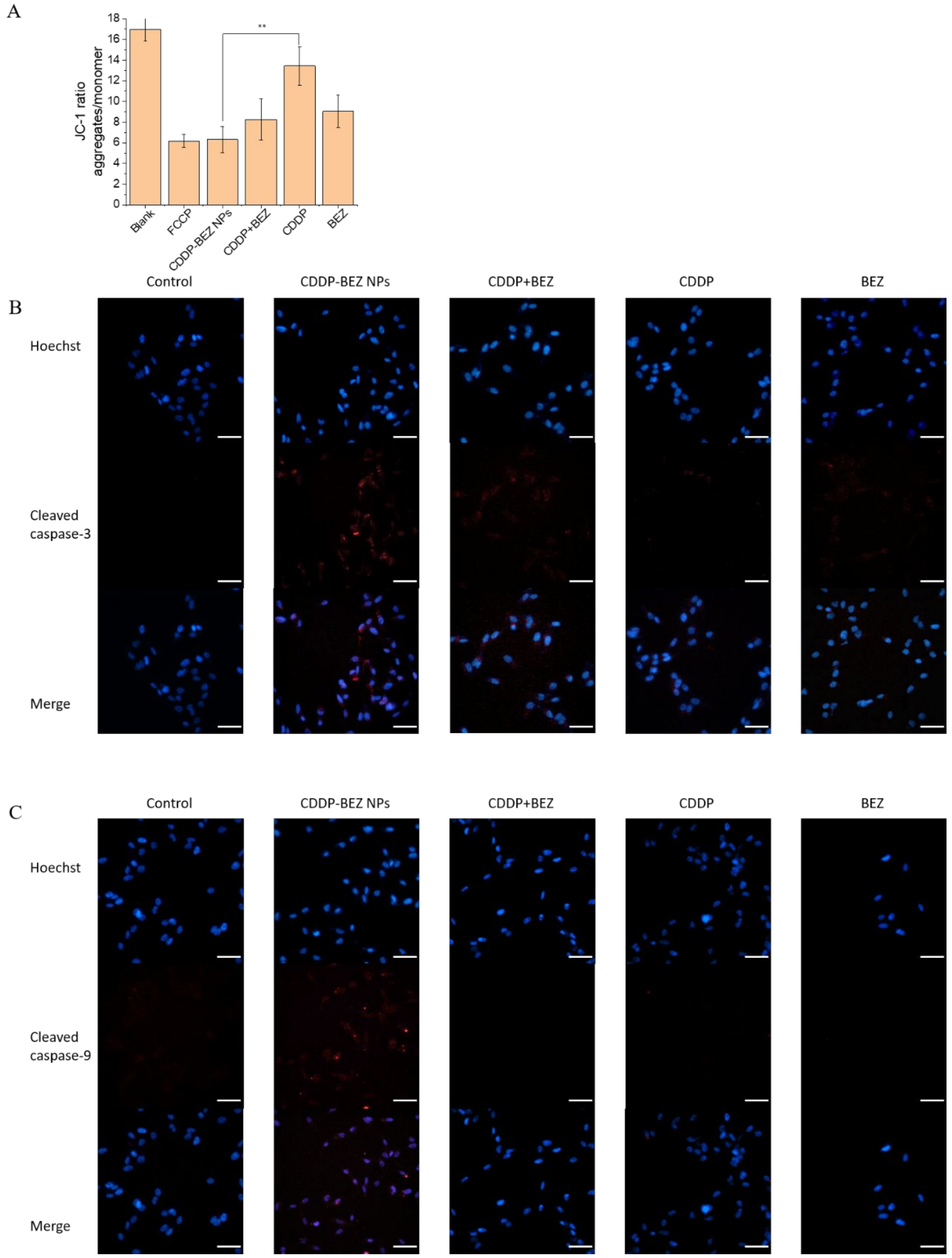

Figure S8. CDDP-BEZ NPs activated intrinsic apoptosis pathway by contributing to mitochondrial depolarization in MDA-MB-231 cells. (A), Mitochondrial potential measured by JC-1 assay in MDA-MB-231 cells treated with different formulations; Immunofluorescence of (B) cleaved caspase-3 and (C) cleaved caspase-9 in MDA-MB-231 cells treated with different formulations (Scale bar = 50 μm).

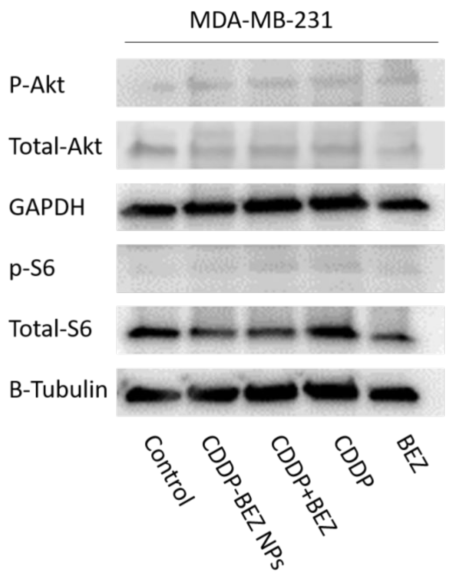

Figure S9. Inhibition on the phosphorylation process of PI3K signaling pathway investigated with western blotting analysis of phosphorylated- or total- AKT and S-6 ribosomal complex in MDA-MB-231 cells.
